# ASNA1 is essential for cardiac development and function by regulating tail-anchored protein stability and vesicular transport in cardiomyocytes

**DOI:** 10.1371/journal.pgen.1011964

**Published:** 2025-12-10

**Authors:** Wei Feng, Zengming Zhang, Zeyu Chen, Li Wang, Mao Ye, Yusu Gu, Titania Huang, Harrison Ngo, Ju Chen

**Affiliations:** 1 Division of Cardiology, Department of Medicine, University of California San Diego, La Jolla, California, United States of America; Geisel School of Medicine at Dartmouth, UNITED STATES OF AMERICA

## Abstract

Recent studies have linked compound heterozygous mutations in ASNA1 to progressive dilated cardiomyopathy and early infantile mortality in humans. However, the specific role of ASNA1 in cardiomyocytes and the molecular mechanisms underlying ASNA1-related cardiomyopathy remain poorly understood. Tail-anchored (TA) proteins, characterized by a single C-terminal transmembrane domain (TMD), require post-translational targeting to intracellular membranes, a process primarily mediated by the evolutionarily conserved Guided Entry of Tail-anchored proteins (GET) pathway in yeast and the Transmembrane Recognition Complex (TRC) pathway in mammals. ASNA1 (also known as TRC40 or GET3) serves as the central ATP-dependent chaperone delivering TA proteins to the endoplasmic reticulum (ER) membrane. To address ASNA1’s role in the heart, we generated constitutive and inducible cardiomyocyte-specific Asna1 knockout mouse models. Constitutive Asna1 deletion during embryogenesis caused perinatal lethality with marked ventricular myocardial thinning by embryonic day 16.5, whereas inducible deletion in adult cardiomyocytes led to rapid ventricular dilation, impaired cardiac function, pathological remodeling, and early mortality. Mechanistically, ASNA1 deficiency destabilized the pre-targeting complex and reduced the expression of multiple TA protein substrates, impairing membrane trafficking and protein transport. Transcriptomic analyses revealed compensatory upregulation of genes involved in protein trafficking and Golgi-to-ER transport, reflecting maladaptive responses to disrupted vesicular transport. Collectively, our findings identify ASNA1 as a critical regulator of TA protein stability and vesicular trafficking in cardiomyocytes, whose loss disrupts cardiac proteostasis and contributes to the cardiomyopathy pathogenesis. Our work provides mechanistic insights into ASNA1-related cardiac disease and highlights potential therapeutic targets.

## Author summary

Cardiomyopathy is a condition characterized by structural and functional abnormalities of the heart muscle, which can impair its ability to pump blood effectively and may eventually lead to heart failure. In this study, we investigated the role of ASNA1, a key protein that delivers other proteins to the endoplasmic reticulum, in the development of cardiomyopathy. ASNA1 has been linked to genetic dilated cardiomyopathy in humans, but its specific function in heart cells was not well understood. We generated mouse models with specific deletions of Asna1 in cardiomyocytes, the cells responsible for heart muscle contraction, to examine its role. Our results showed that when Asna1 was deleted early in development, the mice died shortly after birth with severe heart abnormalities. When we deleted Asna1 in adult cardiomyocytes, the mice developed heart failure rapidly, with significant damage to heart function and structure. The loss of ASNA1 disrupted the stability of essential proteins, leading to defects in protein transport and cellular function. This study highlights the crucial role of ASNA1 in maintaining heart cell function and provides new insights into how its absence can lead to heart disease, potentially opening the door to new diagnostic and therapeutic approaches.

## Introduction

Tail-anchored (TA) proteins are a unique class of integral membrane proteins defined by a single hydrophobic transmembrane domain (TMD) near the C-terminus, which anchors them in the lipid bilayer and serves as a targeting signal [1]. They are distributed across various cellular membranes, including mitochondrial outer membrane, endoplasmic reticulum (ER), Golgi, peroxisomal membrane, and nuclear membranes [2]. Comprising up to 5% of the eukaryotic membrane proteome, TA proteins mediate functionally diverse membrane-associated cellular processes, including intracellular vesicular trafficking, apoptosis, autophagy, lipid biosynthesis, and protein quality control [3–5]. Consequently, mutations in TA proteins are associated with a wide range of diseases, including cancers, immune diseases, liver, and kidney diseases in humans [6–10].

The biogenesis of tail-anchored (TA) proteins poses a significant challenge to cellular proteostasis due to the aggregation-prone nature of their C-terminal transmembrane domains (TMDs) following post-translational release from the ribosome [11]. Without proper handling, these hydrophobic domains are susceptible to misfolding and degradation by protein quality control machinery. Hence, specialized pathways are required to chaperone newly synthesized TA proteins and ensure their accurate membrane insertion. A major, evolutionarily conserved pathway is the Guided Entry of Tail-anchored proteins (GET) pathway in yeast, homologous to the Transmembrane Recognition Complex (TRC) pathway in mammals [1]. This process begins with the capture of nascent TA proteins, commonly by the abundant Hsp70-like chaperone Ssa1 in yeast, which transfers them to the cytosolic co-chaperone Sgt2 (SGTA in mammals). SGTA/Sgt2, selectively recruited to the ribosomes translating TA proteins, shields the hydrophobic TMDs from the aqueous environment. The TA protein is then handed off to a pre-targeting complex—comprising TRC35, UBL4A, and BAG6 in mammals (Get4 and Get5 in yeast)—which facilitates its transfer to ASNA1 (also known as TRC40 in mammals or Get3 in yeast), the central ATP-dependent targeting factor. This pre-targeting complex regulates ASNA1’s ATPase activity and conformational state. In its ATP-bound form, ASNA1 adopts a closed dimeric conformation, forming a hydrophobic groove that securely accommodates the TA protein’s TMD [12]. The ASNA1-TA protein complex is subsequently delivered to the endoplasmic reticulum (ER) membrane, where it engages with the ER-localized TRC receptor complex, composed of tryptophan-rich basic protein (WRB) and calcium-modulating cyclophilin ligand (CAML) in mammals (Get1 and Get2 in yeast). Upon binding of ASNA1 to TRC receptor complex and ATP hydrolysis, WRB/CAML forms a hydrophilic groove within the ER membrane [13], which facilitates TMD insertion by destabilizing the lipid bilayer. Following insertion, ASNA1 undergoes ATP re-binding, promoting its release from the membrane and recycling for subsequent round of targeting [14].

As the central player in the GET pathway, ASNA1 is directly responsible for mediating the ATP-dependent delivery of many TA proteins into the ER membrane, a process critical for maintaining cellular membrane integrity and functionality. Early studies utilized Sec61β and RAMP4 as model substrates, demonstrating their ATP-dependent insertion into the ER membrane through GET3 [15]. Syntaxin 5 (Stx5) and Syntaxin 6 (Stx6), both SNARE proteins involved in vesicular trafficking, critically rely on this pathway in vivo. Their steady-state levels are significantly reduced, and they show aberrant subcellular localization when the TRC pathway is impaired [16–18]. Furthermore, VAPA and VAPB, proteins crucial for membrane contact sites and lipid exchange, were identified as ASNA1 targets, with their steady-state levels affected by pathway impairment [19]. Conversely, some TA proteins (e.g., cytochrome b5 and protein tyrosine phosphatase 1B (PTP1B)) are typically not targeted by the GET/TRC pathway, often utilizing alternative or unassisted insertion routes [1].

Mutations in ASNA1 have been associated with heart diseases. For example, biallelic loss of function variants (V163A/C289W,Q305* heterozygous variants) in ASNA1 results in rapidly progressive cardiomyopathy, acute heart failure, and early lethality in infancy [20]. Mechanistically, these mutations destabilize ASNA1, disrupt its zinc-binding domain, or truncate the C-terminal region, impairing its functionality [20]. In animal models, asna1 deficiency in zebrafish leads to irregular heart shapes, contractility dysfunction, and early lethality [20]. Additionally, Asna1 knockout mice exhibit early embryonic lethality between embryonic day (E) 3.5 and E8.5, underscoring ASNA1’s essential role in mouse heart embryogenesis [21]. However, whether ASNA1 is critical for both embryonic heart development and adult heart function, and whether ASNA1 regulates specific TA proteins critical for cardiomyocyte function remain elusive.

Here, we focus on the role of ASNA1 in mammalian cardiomyocytes, highlighting its impact on cardiac development, function, and adaptive responses to its deletion. By utilizing cardiac specific Asna1 constitutive knockout (cKO) and inducible knockout (icKO), we demonstrate that ASNA1 deficiency disrupts pre-targeting complexes, destabilizes TA proteins, impairs protein trafficking, and alters vesicular transport pathways in embryonic and adult hearts. Our study underscores the essential role of ASNA1 in maintaining cardiac function and offers insights into the development of targeted therapy to address cardiac dysfunction caused by ASNA1 deficiency.

## Results

### Loss of ASNA1 in embryonic cardiomyocytes leads to abnormal heart development in mice

**Fig 1:**
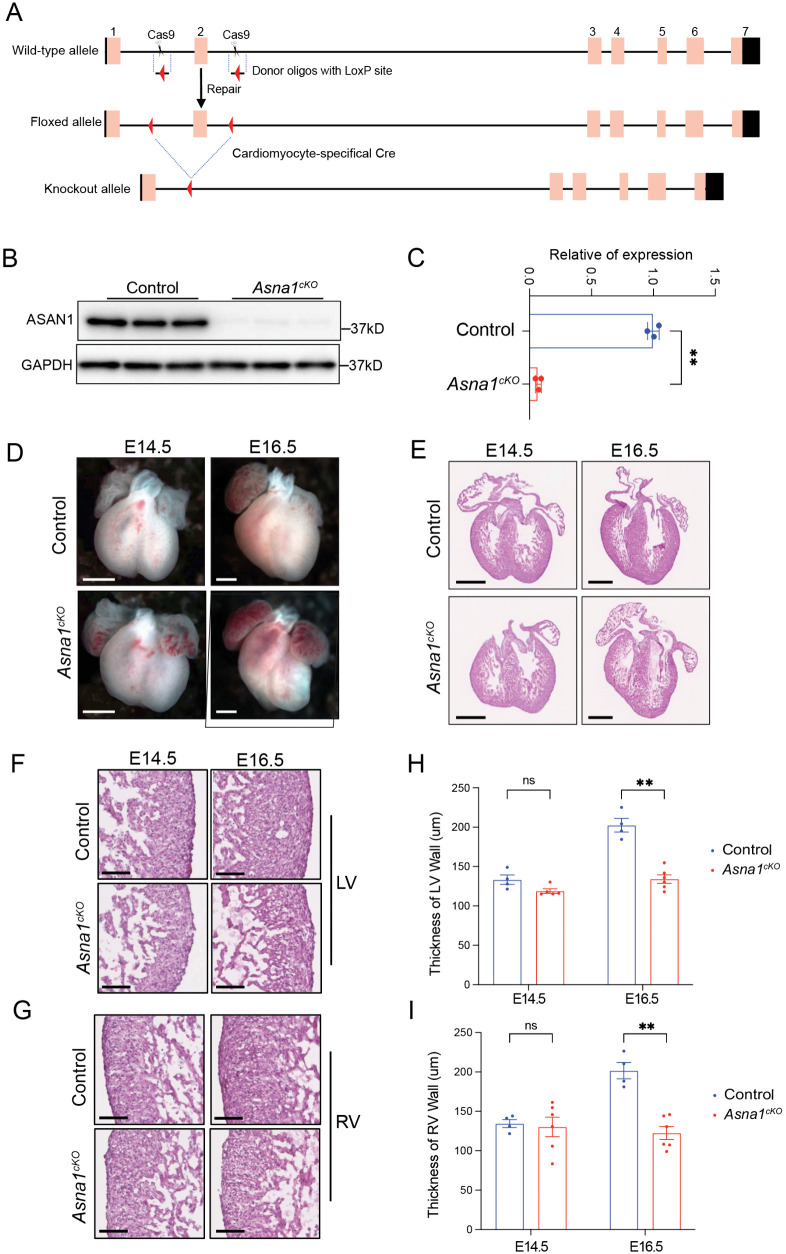
Generation of the Asna1 Floxed Mouse Line and Early Cardiac Structural Defects in Asna1^cKO^ embryonic hearts. **(A)** Schematic illustration of generating the new *Asna1* floxed mice by insertion of two LoxP sites. The LoxP sites are highlighted in red in the donor oligos. **(B-C)** Western blot and corresponding quantitative analysis of ASNA1 protein in E14.5 *Asna1*^*cKO*^ (n = 3 hearts per group) compared to littermate controls. **(D-E)** Representative whole-mount (D) and H&E stained of the whole heart **(E)**, left ventricular (LV) **(F)**, and right ventricular (RV) **(G)** images of E14.5 and E16.5 control and *Asna1*^*cKO*^ mouse hearts (n = 6–8 hearts per group). Scale bar, 0.5 mm **(D-E)**; 0.1mm **(F-G)**. **(H-I)** Measurement of thickness of left ventricular compact myocardium (H) and right ventricular compact myocardium (I) thicknesses on control and *Asna1*^*cKO*^ mice heart sections (n = 3 sections per heart) at E14.5 and E16.5. Data are represented as mean±SD.

To explore the role of ASNA1 in the developing heart, we first constructed an *Asna1* floxed mouse line (*Asna1*^*f/f*^), with LoxP sites flanking the exon 2 of *Asna1* (Fig 1A). Cardiomyocyte (CM)-specific *Asna1* constitutive knockout mice (*Asna1*^*f/f*^; *Xmlc2-Cre*^*+/-*^, hereafter *Asna1*^*cKO*^) were generated by crossing *Asna1*^*f/f*^ with *Xenopus laevis myosin light-chain 2 (Xmlc2)-Cre* mice, which express Cre recombinase specifically in cardiomyocytes as early as embryonic day (E) 7.5 with high efficiency [22–24]. The protein level of ASNA1 was nearly abolished in *Asna1*^*cKO*^ mice hearts, confirming the cardiac specific deletion of ASNA1 (Fig 1B, 1C). *Asna1*^*cKO*^ embryos exhibited striking cardiac abnormalities, including distorted heart morphology and reduced myocardial wall thickness evident by E14.5 and progressively exacerbated by E16.5 (Fig 1D, 1E). These embryos succumbed to perinatal lethality (S1 Table). Hematoxylin and eosin (H&E) staining revealed a markedly thinner compact myocardium in both ventricles at E16.5 in *Asna1*^*cKO*^ hearts (Fig 1F–1I). At E16.5, Ki67 immunostaining revealed a significant reduction in cardiomyocyte proliferation in *Asna1*^*cKO*^ hearts compared to controls (S1A Fig). However, TUNEL staining did not show an increase in apoptosis at this stage (S1B Fig). These data suggest that ASNA1 is critical for normal embryonic heart development.

### ASNA1 loss impairs TRC pathway and disrupts tail-anchored protein trafficking in embryonic cardiomyocytes

**Fig 2:**
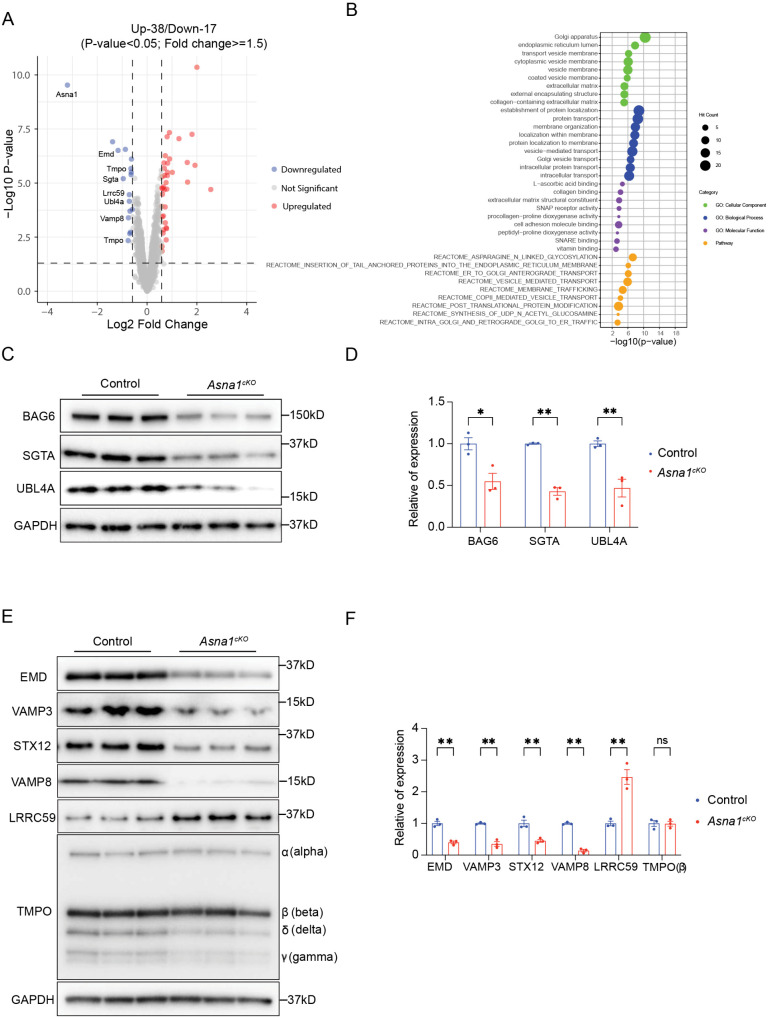
Global Proteomic Changes and Validation of Tail-anchored Protein Targeting Defects in Asna1^cKO^ embryonic hearts. **(A)** Volcano plot showing the differential protein expression between Control and *Asna1*^*cKO*^ hearts at E14.5 (n = 4 hearts per group). Proteins with significant changes (adjusted p-value < 0.05 and log2 fold change > ±1.5) are highlighted in red (upregulated) and blue (downregulated), respectively, while nonsignificant proteins are shown in gray. The dashed lines indicate the significance thresholds. **(B)** Gene Ontology (GO) term enrichment analysis of significantly differentially expressed proteins. Different dot colors represent the top enriched GO terms across biological process, molecular function, cellular component, and pathway categories. Terms are ranked by their enrichment scores (-log10 p-value), with dot size indicating the hit count. **(C-F)** Western blot and corresponding quantitative analysis of components in pre-targeting complexes or six predicted TA protein substrates in E14.5 *Asna1*^*cKO*^ (n = 3 hearts per group) compared to littermate controls. Data are represented as mean±SD.

As ASNA1 is critical for regulating the membrane-insertion of TA proteins, we aimed to investigate if the loss of ASNA1 impairs the proteostasis of certain TA proteins in embryonic cardiomyocytes, leading to abnormal cardiac development. When the transmembrane recognition complex (TRC) pathway is compromised, TA proteins often exhibit reduced abundance due to inefficient membrane insertion, mislocalization, and subsequent degradation by protein quality control mechanisms. We performed proteomic analysis on ventricular tissues isolated from *Asna1*^*cKO*^ and littermate control mice at embryonic day 14.5 (E14.5), prior to the onset of overt cardiac abnormalities. Using a significance threshold of p < 0.05 and a fold change ≥ 1.5 of protein abundance quantified by label-free quantification (LFQ), we identified a total of 55 differentially expressed proteins in the hearts of *Asna1*^*cKO*^ mice, of which 38 proteins were significantly upregulated and 17 proteins were significantly downregulated compared to littermate control (Fig 2A). Gene ontology (GO) enrichment analysis revealed that the differentially expressed proteins in *Asna1*^*cKO*^ hearts were primarily associated with membrane trafficking and protein transport pathway (Fig 2B), consistent with the established role of ASNA1 in the trafficking of tail-anchored (TA) proteins. Our analysis also identified “insertion of tail-anchored proteins into the endoplasmic reticulum membrane” as one of the top enriched pathways among the differentially expressed proteins in *Asna1*^*cKO*^ hearts (Fig 2B). Indeed, label-free quantification of TRC pathway components—including the pre-targeting complex proteins SGTA, BAG6, and UBL4A—revealed their decreased levels in *Asna1*^*cKO*^ hearts compared to controls. This reduction was further confirmed by western blotting (Fig 2C, 2D). Interestingly, qRT-PCR analysis showed no significant changes in mRNA levels of these components in *Asna1*^*cKO*^ hearts (S2A Fig), indicating that ASNA1 loss likely destabilizes the pre-targeting complex at the protein level within the TRC pathway. Additionally, our proteomics analysis revealed significant alteration in five predicted tail-anchored proteins (EMD, VAMP3, STX12, VAMP8 and LRRC59) in *Asna1*^*cKO*^ hearts [25,26]. Western blotting confirmed decreased protein levels of VAMP3, STX12, VAMP8, and EMD (Fig 2E, 2F). Interestingly, none of these showed changes in mRNA levels in *Asna1*^*cKO*^ hearts as assessed by qRT-PCR (S2B Fig). In parallel, both the mRNA level and protein level of LRRC59 were elevated in *Asna1*^*cKO*^ heart (Figs 2E, 2F and S2B), suggesting that this increase may be secondary to the abnormal heart development.

In summary, our findings indicate that ASNA1 is essential for maintaining the stability and function of the pre-targeting complex and its TA protein substrates. Consequently, loss of ASNA1 poses significant implications for cellular proteostasis and plays potential roles in the pathophysiology of cardiac diseases.

### Enhanced ER-Golgi transport of COPI and COPII vesicles following ASNA1 deletion

**Fig 3:**
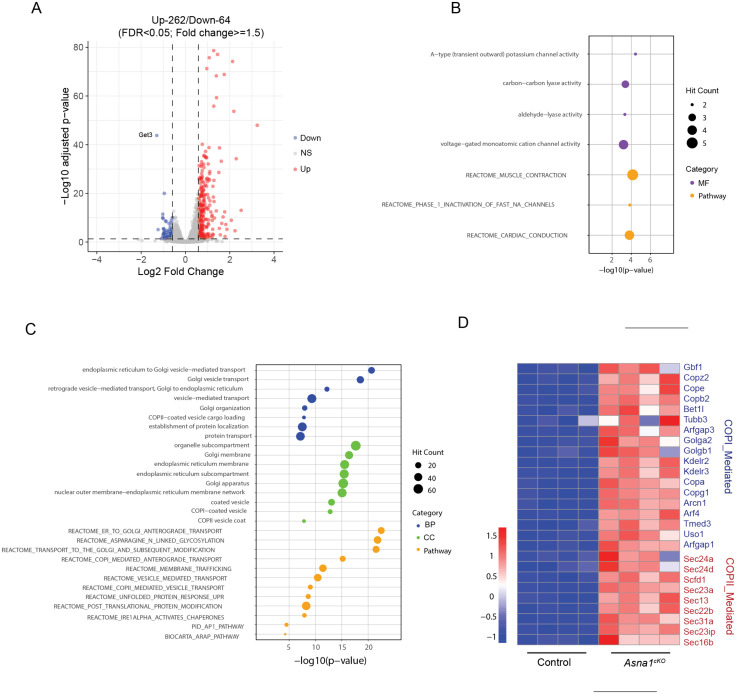
Global Transcriptomic Changes and Downstream Vesicle Transport Defects in Asna1^cKO^ embryonic hearts. **(A)** Volcano plot of differentially expressed genes (DEGs) between control and *Asna1*^*cKO*^ ventricles at E14.5 (n = 4 hearts per group). DEGs with adjusted P < 0.05 and fold change ≥ 1.5 are considered significantly upregulated (red dots) or downregulated genes (blue dots), while nonsignificant genes are shown in gray. **(B-C)** Gene ontology (GO) analysis of downregulated (B) and upregulated **(C)** DEGs in *Asna1*^*cKO*^ ventricles at E14.5. The top enriched GO terms are shown, categorized by biological process, molecular function, cellular component or pathway, with dot size indicating the hit count. **(D)** Heatmap illustrating the expression levels of COPI- and COPII-mediated vesicles transport proteins in *Asna1*^*cKO*^ and control hearts at E14.5. Expression values are normalized, with higher expression shown in red and lower expression in blue.

To further investigate how ASNA1 ablation in cardiomyocytes (CMs) affects cellular processes, we performed RNA-seq on ventricular tissue from *Asna1*^*cKO*^ and littermate control hearts at embryonic day 14.5 (E14.5). Using an adjusted p-value threshold of < 0.05 and a fold change ≥ 1.5, we identified 326 differentially expressed genes (DEGs), including 64 downregulated and 262 upregulated genes in *Asna1*^*cKO*^ hearts compared to the littermate controls (Fig 3A).

Gene Ontology (GO) and Reactome pathway analyses were conducted to elucidate the biological processes altered by ASNA1 deletion. Among the downregulated genes, GO terms were significantly enriched for ion channel activity, particularly A-type (transient outward) potassium channels and voltage-gated monovalent cation channels (Fig 3B). Reactome analysis supported these findings, showing downregulation in pathways related to muscle contraction and cardiac conduction, indicating potential impairments in cardiomyocyte contractility and electrophysiological function. Conversely, the upregulated DEGs were significantly enriched in pathways related to vesicle-mediated transport (Fig 3C, 3D). Genes in GO terms including “endoplasmic reticulum to Golgi vesicle-mediated transport”, “Golgi vesicle transport”, and “retrograde vesicle-mediated transport, Golgi to ER” were among the most significantly overrepresented. Additionally, cellular component terms such as Golgi membrane, ER membrane, and COPI- and COPII-coated vesicles were also enriched, indicating altered trafficking between the ER and Golgi compartments. Reactome pathway analysis further highlighted enrichment in “ER to Golgi anterograde transport,” “membrane trafficking,” and vesicle formation pathways involving COPI and COPII vesicles.

The concurrent dysregulation of multiple TA proteins in *Asna1*^*cKO*^ hearts, including EMD, VAMP3, STX12, VAMP8 and LRRC59, suggests that the enrichment of trafficking pathways reflects a compensatory transport network. Specifically, the loss of ASNA1 likely impairs the stability and membrane integration of key TA proteins, leading to defective vesicle formation and cargo delivery. In addition, proteomic analysis also revealed increased protein abundance in vesicle trafficking components (Fig 3C, 3D), further indicating system-wide dysregulation of the ER-Golgi transport axis due to impaired TA protein biogenesis in the absence of ASNA1.

### *ASNA1* ablation in CMs does not activate endoplasmic reticulum stress

**Fig 4:**
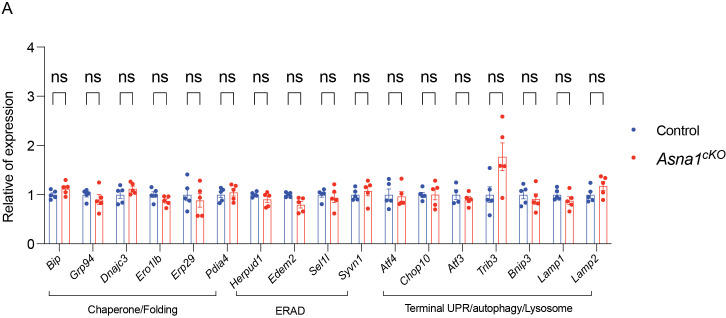
Quantitative PCR analysis of ER stress and autophagy-related gene expression in *Asna1*^*cKO*^ embryonic hearts. **(A)** Relative mRNA expression levels of genes involved in chaperone/folding (Bip, Grp94, Dnajc3, Ero1lb, Erp29, Pdia4), ER-associated degradation (ERAD; Herpud1, Edem2, Sel1l, Syn1), and terminal UPR/autophagy/lysosome pathways (Atf4, Chop10, Atf3, Trib3, Bnip3, Lamp1, Lamp2) were assessed by qPCR in E14.5 embryonic hearts from control (blue) and *Asna1*^*cKO*^ (red) mice. Expression values were normalized to housekeeping genes and are shown relative to control. Each dot represents an individual biological replicate; bars indicate mean ± SD. No statistically significant differences were observed between groups (ns, not significant).

From the above data, ASNA1 deletion impairs the stability and functionality of the components of the pre-targeting complexes and TA proteins, leading to an adaptive response within the ER and Golgi transport in CMs. This could result in ER stress and activation of the unfolded protein response (UPR) in heart development. Previous studies have highlighted ASNA1’s critical role in protein targeting and its involvement in ER stress activation upon knockout [16,18]. To test this hypothesis, we first examined the markers of ER stress, unfolded protein response (UPR), ER-associated degradation (ERAD), and autophagy/lysosome functions using RNA-seq data at E14.5. Surprisingly, our analysis revealed no significant changes in the expression of these genes in *Asna1*^*cKO*^ hearts compared to the controls (S3 Fig). To exclude the possibility that ER stress activation occurs at a later development stage, we next assessed these pathways at the E16.5 stage using qRT-PCR. Consistent with the E14.5 findings, there were no significant differences in the expression of key genes involved in ER stress, UPR, ERAD, or autophagy/lysosome functions between control and *Asna1*^*cKO*^ heart (Fig 4A). These results indicate that the loss of ASNA1 does not lead to the immediate activation of ER stress or related adaptive pathways during the early to mid-stage of heart development.

### Rapid myocardial dysfunction with robust remodeling following *Asna1* deletion in adult CMs

**Fig 5:**
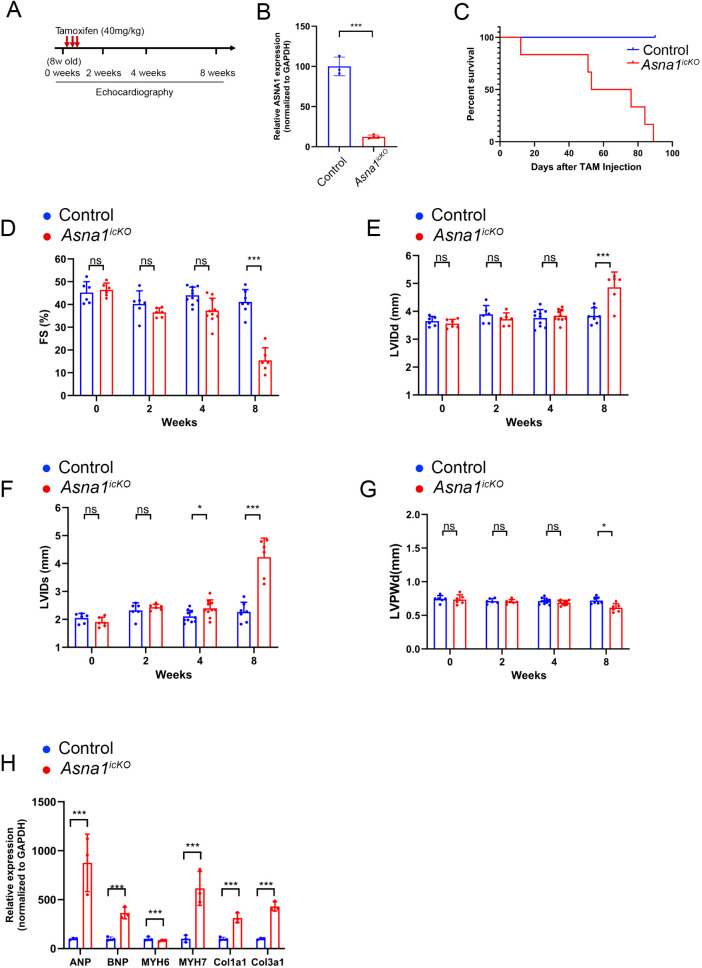
Rapid deterioration of cardiac function following cardiomyocyte-specific *ASNA1* deletion. **(A)** Tamoxifen administration schedule. Red arrows indicate the days of tamoxifen injection. Echocardiography was performed on *Asna1*^*f/f*^ (control) and inducible cardiomyocyte-specific knockout (*Asna1*^*f/f*^:*MerCreMer + , Asna1*^*icKO*^) mice at baseline (Day 0) and at 2, 4, and 8 weeks post-injection. **(B)** Kaplan–Meier survival curves for control (blue) and *Asna1*^*icKO*^ (red) mice. Survival was monitored weekly following tamoxifen administration (*n* = 6 for control, *n* = 13 for ICKO). **(C–G)** Representative echocardiographic measurements of control and *Asna1*^*icKO*^ mice at baseline (Day 0) and at 2, 4, and 8 weeks post-injection. **(H)** RT-qPCR analysis of cardiac remodeling and fibrosis markers in control and *Asna1*^*icKO*^ mice at 4 weeks post-injection (N = 3 per group). Abbreviations: FS, fractional shortening; LVIDd, left ventricular internal diameter at diastole; LVIDs, left ventricular internal diameter at systole; LVPWd, left ventricular posterior wall thickness at diastole. Data are presented as mean ± individual values. Statistical analysis was performed using two-way ANOVA followed by Tukey’s multiple comparison test. *P* < 0.01 (*), *P* < 0.001 (***).

To investigate the role of ASNA1 in the adult heart, we employed a MerCreMer-mediated inducible Cre-LoxP system to delete Asna1 in 8-week-old mice following tamoxifen administration (Fig 5A) [27,28]. Upon tamoxifen binding, MerCreMer translocates from the cytoplasm to the nucleus, where it mediates recombination of floxed alleles. By three weeks after the initial tamoxifen injection, approximately 80% of Asna1 mRNA was depleted in inducible cardiomyocyte-specific knockout (*Asna1*^*icKO*^) hearts (Fig 5B), demonstrating the efficiency of the inducible knockout of ASNA1. All *Asna1*^*icKO*^ mice died within three months of tamoxifen injection, with a median survival of 50 days (Fig 5C).

Cardiac function was evaluated using M-mode echocardiography at serial time points, assessing fractional shortening and left ventricular systolic/diastolic dimensions (Fig 5D–5G). Tamoxifen-treated *Asna1*^f/f^; MCM-Cre^+^ (*Asna1*^*icKO*^) mice consistently exhibit significant reductions in fractional shortening and prominent ventricular dilation by 8 weeks post tamoxifen injection (Fig 5D–5F), along with decreased wall thickness (Fig 5G), suggesting the development of progressive dilated cardiomyopathy. Quantitative PCR analysis revealed a significant upregulation of cardiac stress markers, including *Nppa* (ANP), *Nppb* (BNP), *Myh6*, *Myh7*, *Col1a1*, and *Col3a1* genes, in *Asna1*^*icKO*^ hearts compared to controls, indicative of extensive cardiac remodeling (Fig 5H). These data suggest that ASNA1 is essential for adult cardiac function in addition to embryonic cardiac development.

### ASNA1 Deletion Downregulates the expression of Pre-Targeting Complex components and a Subset of Tail-Anchored Proteins in the Adult Heart

To assess the molecular consequences of ASNA1 deficiency on the TRC pathway in adult hearts, we performed Western blot analysis to examine the expressions of key components involved in TA protein targeting and membrane insertion. These included the cytosolic pre-targeting machinery components—SGTA, UBL4A, and BAG6—as well as the central targeting factor ASNA1 and the ER-localized TRC receptor components WRB and CAML.

**Fig 6:**
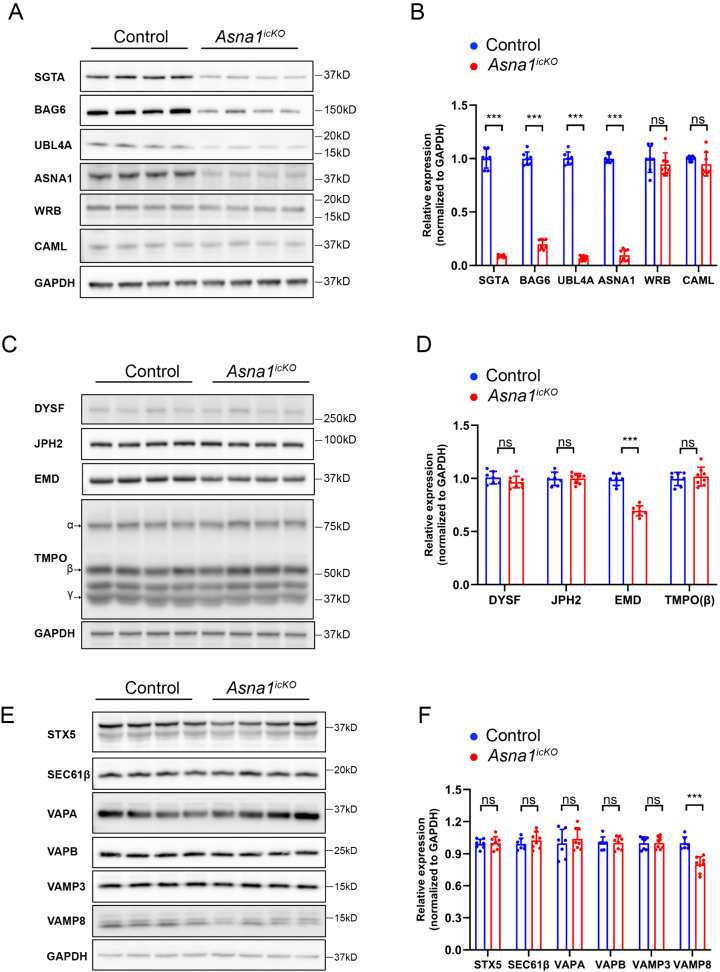
Expression of TRC pathway components and tail-anchored (TA) proteins in adult cardiomyocyte-specific ASNA1 knockout (*Asna1*^*icKO*^) hearts. **(A)** Western blot analysis of key TRC pathway components in heart lysates from *Asna1*^*icKO*^ and control mice. GAPDH was used as a loading control. **(B)** Densitometric quantification of protein levels shown in **(A)**. **(C)** Western blot analysis of selected cardiomyopathy-related TA proteins in the same samples, with GAPDH as a loading control. **(D)** Quantification of TA protein expression levels shown in **(C)**. **(E)** Additional TA proteins were assessed by western blot using GAPDH as a loading control. **(F)** Quantification of the western blot data shown in **(E)**.

Hearts from tamoxifen-treated Asna1 inducible cardiomyocyte-specific knockout (*Asna1*^*icKO*^) and control mice were harvested at 4 weeks post tamoxifen induction, and total protein lysates were subjected to immunoblotting. As expected, the protein level of ASNA1 was markedly reduced in the *Asna1*^*icKO*^ hearts, confirming efficient gene deletion (Fig 6A, 6B). Interestingly, expressions of components of the pre-targeting complex, including SGTA, UBL4A, and BAG6, were also significantly decreased, suggesting that ASNA1 may stabilize or regulate the homeostasis of the cytosolic pre-targeting complex. In contrast, the levels of WRB and CAML, the two essential subunits of the ER-localized TRC receptor complex, remained unchanged, indicating that ASNA1 deficiency selectively affects upstream components of the TRC pathway.

We next examined whether ASNA1 deficiency alters the expression of cardiomyopathy-related TA proteins. Western blot analysis was performed on left ventricular lysates from control and *Asna1*^*icKO*^ mice at 4 weeks post-tamoxifen administration, focusing on DYSF, EMD, JPH2, and TMPO(β). Among these, EMD, a nuclear envelope-associated TA protein involved in nuclear-cytoskeletal coupling, was modestly but significantly reduced in *Asna1*^*icKO*^ hearts (Fig 6C, 6D). In contrast, protein levels of DYSF, JPH2, and TMPO(β) were not significantly altered.

We further evaluated the expression of additional TA proteins to determine whether ASNA1 loss differentially affects TA protein stability. Vesicle-associated membrane protein 8 (VAMP8), a vesicular SNARE involved in endocytic and secretory vesicle fusion and was downregulated in embryonic *Asna1*^*cKO*^ hearts, was significantly reduced in *Asna1*^*icKO*^ hearts compared to controls. However, the steady-state levels of Sec61β (an auxiliary subunit of the translocon), syntaxin 5 (Stx5), two TA proteins that have shown to be dependent on TRC pathway in mammals [16,17], were not significantly altered (Fig 6E, 6F). Other TA proteins such as VAPA and VAPB, which have been identified as target substrates of TRC40 in human cells [19] and VAMP3, which was downregulated in embryonic *Asna1*^*cKO*^ hearts, were not significantly affected (Fig 6E, 6F).

Together, these results indicate that ASNA1 deficiency leads to selective destabilization of the pre-targeting complex and a subset of functionally important TA proteins in adult hearts, potentially contributing to impaired vesicular trafficking and nuclear envelope function.

## Discussion

In this study, we demonstrate a critical physiological role of ASNA1 in regulating the stability of pre-targeting complex and vesicular transport of a subset of tail-anchored (TA) proteins in cardiomyocytes. Deletion of *Asna1* in embryonic cardiomyocytes resulted in perinatal lethality and pronounced ventricular myocardial thinning by embryonic day 16.5. Inducible *Asna1* deletion in adult cardiomyocytes caused rapid-onset ventricular dilation, impaired cardiac function, pathological remodeling, and early mortality. These results suggest that ASNA1 is essential for both embryonic cardiac development and adult cardiac function.

To determine if ASNA1 regulates embryonic cardiac development and cardiac function in adults by regulating the stability and trafficking of specific TA proteins, we performed proteomic analysis in *Asna1*^*cKO*^ hearts, as well as assessed the levels of candidate TA proteins in *Asna1*^*icKO*^ hearts. We show that ASNA1 loss in embryonic hearts impairs the TRC pathway and disrupts TA protein trafficking, with TA proteins EMD, VAMP3, STX12 and VAMP8 being significantly downregulated upon ASNA1 loss. In parallel, ER-to-Golgi transport via COPI and COPII vesicles is enhanced, suggesting compensatory remodeling of vesicular trafficking pathways. Interestingly, assessment of candidate TA protein expression in adult *Asna1*^*icKO*^ hearts shows only the downregulation of VAMP8 and EMD. While these findings support a role for ASNA1 in regulating trafficking of a subset of TA proteins, they are based on expression data and remain correlative. Further functional studies will be needed to confirm direct mechanistic links.

Our findings demonstrate a context-dependent role for ASNA1 in regulating the trafficking and stability of TA proteins, reflecting differential dependencies in embryonic versus adult hearts. For example, five TA proteins—EMD, VAMP3, STX12, VAMP8 and LRRC59—are dysregulated in Asna1 conditional knockout (*Asna1*^*cKO*^) embryonic hearts. In contrast, only two of these proteins, EMD and VAMP8, are downregulated in adult hearts lacking ASNA1, while the levels of VAMP3, STX12, LRRC59, and TMPO remain unchanged. These data suggest context-specific requirements for ASNA1 in maintaining TA protein homeostasis, but additional experiments are needed to validate the functional significance of individual TA protein changes. Importantly, mutations in EMD have been previously linked to cardiomyopathy [29,30], highlighting the clinical relevance of their dysregulation in this context.

Indeed, although the primary route for tail-anchored (TA) proteins to reach the endoplasmic reticulum (ER) is the evolutionarily conserved guided entry of TA proteins (GET) pathway in yeast and its mammalian counterpart, the transmembrane recognition complex (TRC) pathway, alternative targeting mechanisms also exist. These include an unassisted pathway for moderately hydrophobic TA proteins and several chaperone-mediated routes, such as those involving Hsc70/Hsp40, the signal recognition particle (SRP) functioning in a post-translational mode, as well as the recently identified SRP-independent targeting (SND) pathway and the ER membrane complex (EMC) pathway. Together, these pathways constitute a complex and partially redundant network for TA protein biogenesis [31,32].

Nascent tail-anchored (TA) proteins are initially captured by the cytosolic co-chaperone SGTA to prevent aggregation caused by their hydrophobic transmembrane domain (TMD). Subsequently, the pre-targeting complex—comprising TRC35, UBL4A, and BAG6—facilitates the handoff of TA proteins from SGTA to ASNA1. Structurally, BAG6 serves as the central scaffold of this complex [33]. TRC35 and UBL4A bind distinct motifs within the C-terminal region of BAG6, forming a two-armed configuration. UBL4A, which contains a ubiquitin-like (UBL) domain, recruits SGTA, while TRC35 interacts with ATP-bound ASNA1 in a nucleotide-dependent manner. Although the endoplasmic reticulum (ER) receptor subunits WRB and CAML display mutual interdependence for stability and biogenesis [34,35], knockout mouse models of individual pre-targeting complex components (TRC35, UBL4A, BAG6) have not revealed similar interdependence among themselves. Previous studies using Cre/loxP-mediated tissue-specific knockout of Asna1 in pancreatic β-cells and progenitor cells also did not examine how disruption of ASNA1 affects the integrity of the pre-targeting complex [16,18]. The regulatory mechanisms governing expression of the pre-targeting complex in vivo remain poorly understood.

In our cardiomyocyte-specific Asna1 knockout models (both constitutive and inducible), we demonstrate for the first time that ASNA1 is essential for stabilizing the pre-targeting complex in embryonic and adult cardiomyocytes. Interestingly, despite unchanged mRNA levels, TRC35, UBL4A, and BAG6 proteins are significantly reduced in ASNA1-deficient hearts, suggesting that ASNA1 post-translationally regulates the pre-targeting complex stability. Mechanistically, TRC35 preferentially binds ATP-bound ASNA1, locking it into an “occluded” state that inhibits premature ATP hydrolysis and primes it for substrate capture [36]. While our data support a model in which ASNA1 stabilizes the pre-targeting complex through post-translational mechanisms, this conclusion is primarily based on protein expression patterns and will require further validation through direct functional analysis. Our findings indicate that in the absence of ASNA1, the two-armed configuration of the pre-targeting complex might become destabilized, leading to its impaired assembly and increased susceptibility to proteolytic degradation. These results reveal a previously unrecognized post-translational role for ASNA1 in maintaining the structural integrity and functional stability of the pre-targeting complex in cardiomyocytes. Whether this mechanism is conserved in other cell types, tissues, or physiological contexts remains to be fully investigated.

While our study establishes that ASNA1 is essential for maintaining the stability of the TRC pre-targeting complex components (SGTA, BAG6, UBL4A) in cardiomyocytes, the precise biochemical mechanisms underlying this stabilization remain to be elucidated. Previous studies have demonstrated functional and physical interactions among these factors, with SGTA directly binding to BAG6 and UBL4A to form a triage complex for tail-anchored proteins [37,38]. ASNA1/TRC40 interacts with SGTA and facilitates ATP-dependent substrate transfer, suggesting that ASNA1 may stabilize the complex through direct binding or conformational regulation driven by its ATPase cycle [12,39]. However, it is currently unclear whether ASNA1 protects these components from ubiquitination and proteasomal degradation or if stabilization occurs indirectly via modulation of complex assembly or turnover. Our data do not directly address whether ASNA1 physically shields the pre-targeting factors or regulates their post-translational modifications. Further biochemical studies, including co-immunoprecipitation, binding assays, and analysis of ATPase-deficient ASNA1 mutants, will be required to dissect these mechanistic aspects in detail.

In summary, our findings indicate that ASNA1 is essential for maintaining the stability and function of the pre-targeting complex and its TA protein substrates. Consequently, loss of ASNA1 poses significant implications for cellular proteostasis and plays potential roles in the pathophysiology of cardiac diseases.

## Materials and methods

### Ethics statement

All animal procedures were performed in accordance with the National Institutes of Health Guide for the Care and Use of Laboratory Animals and approved by the Institutional Animal Care and Use Committee of the University of California San Diego with approved protocol # S01049.

### Mice

The Asna1 floxed mouse line was generated using CRISPR-Cas9 technology, as previously described [40]. Briefly, Cas9 protein, crRNA, tracrRNA, and single-stranded oligodeoxynucleotide (ssODN) repair templates were mixed in IDTE buffer and microinjected into the pronuclei of C57BL/6N zygotes. The crRNA sequences used were 5’-acuucuauuugaugccagagGUUUUAGAGCUAUGCUGUUUUG-3’ (crRNA-L) and 5’-ggaaggaaaccuuugaggagGUUUUAGAGCUAUGCUGUUUUG-3’ (crRNA-R), and the ssODN sequences were 5’- AGTAGAGGGAGCGACACTAAGGCAAGCTGTCTTAGTATAACAGACTTCTATTTGATGCCA**ATAACTTCGTATAGCATACATTATACGAAGTTAT**GAGAGGGAAGAAGTAAAACGGAGTGGAAAGGGCACGTGATTATGGTGCCTCGGTTCGTTG-3’ (ssODN-L) and 5’- CTCTAGGCGTCACACCCCCAACATACACCCAGAGAGGCTTGTGGGAAGGAAACCTTTGAG**ATAACTTCGTATAGCATACATTATACGAAGTTAT**GAGCGGGGAGAAAGGCGATAAACTGGAACAGAGCAAACAAGAAGGAGGCCCAACCAGAGA-3’ (ssODN-R), with LoxP sites indicated in bold. Zygotes were then transferred into the oviductal papillae of pseudopregnant ICR female mice. Genomic DNA was extracted from offspring, and successful insertion of LoxP sites at the Asna1 locus was confirmed by PCR and direct sequencing.

Cardiomyocyte-specific Asna1 knockout mice were generated by crossing *Asna1*^*f/f*^ mice with Xmlc2-Cre mice [22], and tamoxifen-inducible cardiac-specific knockouts were produced by crossing *Asna1*^*f/f*^ mice with αMHC-MerCreMer transgenic mice (Jackson Laboratory, Farmington, CT, USA).

Mouse genotyping was performed by PCR using genomic DNA extracted from embryonic yolk sacs or adult tail tissue. Primer sets used included: Asna1 (forward: 5’-TGGAGGTTGTAGGATCCCAG-3’; reverse: 5’-ATGTTAGTAGTCAGGGAGAGGC-3’), Asna1 knockout allele (forward: 5’-GCCCTGTGAGCTCCATGTATC-3’; reverse: 5’-TCAATGTTCGTAAAATTGATTAACAAGC-3’), Asna1 floxed allele (forward: 5’-GGACGTACCTACCAGCAGCCAC-3’; reverse: 5’-CCAAACTGCTTGCCCAGCCAGC-3’), and Cre (forward: 5’-TGCCTGCATTACCGGTCGATGC-3’; reverse: 5’-CCATGAGTGAACGAACCTGGTCG-3’).

### Western blots

Western blotting was performed as previously described [28,41,42]. Briefly, embryonic or adult mouse hearts were dissected and snap-frozen in liquid nitrogen. Total protein extracts were obtained by homogenizing heart tissue in RIPA buffer. For embryonic hearts, homogenization was carried out using a handheld pellet pestle (Sigma-Aldrich), while adult hearts were processed using a Powergen 700 homogenizer (Fisher Scientific). Protein samples were resolved on Bolt 4–12% Bis-Tris gels (Life Technologies) and transferred to PVDF membranes (Bio-Rad). Membranes were blocked, incubated with primary antibodies overnight at 4°C, and subsequently washed with TBST. Afterward, membranes were incubated with HRP-conjugated secondary antibodies and visualized using enhanced chemiluminescence (ECL) reagent (Bio-Rad). Signals were detected using the Bio-Rad ChemiDoc Imaging System. A complete list of antibodies and catalog numbers used in this study is provided in S2 Table.

### Proteomics sample preparation and mass spectrometry analysis

Proteomic analysis was performed as previously described [28,41] using E14.5 heart lysates processed by the SBP Proteomics Core. Proteins were quantified by BCA assay, reduced with 5 mM TCEP, alkylated with 15 mM IAA, and digested overnight with Trypsin/Lys-C. Peptides were acidified, desalted using C18 cartridges (AssayMap Bravo, Agilent), dried, and reconstituted in 2% ACN/0.1% FA. Concentrations were estimated using a NanoDrop spectrophotometer (Thermo Fisher). LC-MS/MS was conducted using an EASY-nanoLC system coupled to an Orbitrap Fusion Lumos mass spectrometer. Peptides were separated on a C18 Aurora column over a 75-minute gradient at 300 nL/min. MS1 scans were acquired at 60,000 resolution; top precursors (charge +2 to +7) were fragmented by HCD and analyzed in the ion trap. Data were processed with MaxQuant (v1.6.11.0) using the Mus musculus UniProt database with a 1% FDR at the peptide and protein levels. Mass spectrometry data analysis was conducted according to the DEqMS workflow [43]. Volcano plots were generated using the ggplot2 package in R. Gene Ontology (GO) enrichment analysis was performed using ToppGene, and results were visualized with ggplot2. Heatmaps were created using the pheatmap package in R. The mass spectrometry proteomics data generated in this study have been deposited in the MassIVE repository under accession number MSV000098765 and are accessible via https://doi.org/10.25345/C5959CM6Z.

### RNA extraction, library preparation, and RNA-seq analysis

RNA-seq analysis was performed as previously described [44]. Total RNA was extracted from E14.5 mouse ventricular hearts using TRIzol (Invitrogen), and RNA integrity was confirmed (RIN 6.1–10) with an Agilent 2100 Bioanalyzer. RNA-seq libraries were prepared using the Illumina TruSeq Stranded mRNA Library Prep Kit and sequenced (150 bp paired-end) on a HiSeq 4000 platform at the UC San Diego IGM core, yielding ~20–70 million reads per sample. Reads were trimmed with Trim Galore (v0.6.10), quality-checked using FastQC (v0.11.9), and aligned to the mm10 genome (UCSC) using HISAT2 (v2.2.1), with sorting by SAMtools (v1.3.1). Gene counts were generated with featureCounts (v2.0.1) and transcript abundance estimated using StringTie (v2.2.1). Differential expression analysis was performed in R (v4.2.1) using DESeq2, with significance defined as FDR < 0.05 and |fold change| > 1.5. Volcano plots were generated using the ggplot2 package in R. Gene Ontology (GO) enrichment analysis was performed using ToppGene, and results were visualized with ggplot2. Heatmaps were created using the pheatmap package in R. RNA-seq data are available in the Gene Expression Omnibus (GEO) under accession number GSE304685.

### Quantitative real-time polymerase chain reaction

Total RNA was extracted from E14.5 frozen embryonic hearts using TRIzol solution (Invitrogen) following the manufacturer’s protocol. Approximately 1µg of total RNA was used for reverse transcription with M-MLV Reverse Transcriptase according to the manufacturer’s instructions (Promega, M1701). Quantitative PCR was carried out with iTaq Universal SYBR Green Supermix (Bio-Rad, 1725120) on CFX Opus 96 Real-Time Polymerase Chain Reaction System (Bio-Rad). Relative gene expression levels were calculated using the comparative CT method (ΔΔCt method) and normalized to *Polr2a* as the endogenous reference. Primer sequences are listed in S3 Table.

### Echocardiography

Mouse echocardiography was performed as previously described [45]. Briefly, mice were initially anesthetized with 5% isoflurane (VETone, 502017) for 1 minute and maintained under 1% isoflurane throughout the imaging procedure. The anterior chest area was shaved, and any remaining hair was removed. For simultaneous electrocardiogram monitoring, small needle electrodes were inserted into one upper and one lower limb. Transthoracic echocardiography (M-mode and 2-dimensional) was performed using the VisualSonics Vevo 2100 ultrasound system equipped with a linear transducer 32–55MHz. Cardiac parameters including heart rate (HR), left ventricular end-diastolic dimensions (LVEDD) and left ventricular end-systolic dimensions (LVESD), end-diastolic interventricular septal thickness (IVSd) and LV posterior wall thickness (LVPWd) were measured from the M-mode images. Fractional shortening (%FS) was calculated and used as an indicator of systolic cardiac function.

### Statistical analysis

Image analysis was performed using ImageJ software. Statistical analyses were conducted using GraphPad Prism 8. Data are presented as mean ± SD. The Shapiro–Wilk test was used to assess the normality of data distribution. For normally distributed data, comparisons between two groups were conducted using Student’s t-test, and comparisons among multiple groups were performed using one-way ANOVA followed by Tukey’s post hoc test. For non-normally distributed data, the Mann–Whitney U test or Kruskal–Wallis test followed by Dunn’s multiple comparisons test was used, as appropriate. Statistical significance was determined as indicated in the Fig legends. Differences were considered statistically significant at *p* < 0.01 and are denoted as follows: *p* < 0.01 (*), *p* < 0.001 (***).

## Supporting information

S1 Fig Reduced cardiomyocyte proliferation at E16.5 in Asna1 conditional knockout hearts without significant changes in apoptosis. (A) Quantification of Ki67 ⁺ cardiomyocytes (CMs) shows a significant reduction in proliferative cells in *Asna1*^*cKO*^ hearts compared to controls (***, *P* < 0.001). (B) Quantification of TUNEL⁺ cardiomyocytes indicates no significant difference in apoptosis between control and *Asna1*^*cKO*^ groups (ns, not significant). Data are presented as mean ± SD, with individual data points shown.
(PDF)

S2 Fig Quantitative PCR analysis of TRC pathway and TA protein gene expression in *Asna1*^*cKO*^ embryonic hearts. (A) Relative mRNA expression levels of genes involved in the transmembrane recognition complex (TRC) pathway (Asna1, Bag6, Sgta, Ubl4a) were assessed by qPCR in E14.5 embryonic hearts from control (blue) and *Asna1*^*cKO*^ (red) mice. (B) Relative mRNA expression levels of selected tail-anchored (TA) protein-encoding genes (Emd, Vamp3, Stx12, Vamp8, Lrrc59) were measured from control (blue) and *Asna1*^*cKO*^ (red) hearts. Expression values were normalized to housekeeping genes and are shown relative to control. Each dot represents an individual biological replicate; bars indicate mean ± SD.
(PDF)

S3 Fig Heatmap of RNA-seq analysis at E14.5 showing transcript levels of representative genes involved in ER stress, the unfolded protein response (UPR), ER-associated degradation (ERAD), and autophagy–lysosome pathways. No significant differences in gene expression were observed between *Asna1*^*cKO*^ and control hearts.
(PDF)

S1 Table Numbers of control (*Asna1*^*f/f*^ or *Asna1*^*f/+*^), *Asna1*^*cKO*^ (*Asna1*^*f/f*^; *Xmlc2-Cre*^+/-^) and *Asna1*^*cHet*^ (*Asna1*^*f/+*^; *Xmlc2-Cre*^+/-^) from crossing *Asna1*^*f/+*^; *Xmlc2-Cre*^+/-^ males with *Asna1*^*f/f*^ females. (PDF)

S2 Table Antibodies used in this study. (PDF)

S3 Table Primer sequences used for qPCR in this study. (PDF)

S4 Table Numerical data underlying Figs 1C, 1H, 1I, 2B, 2D, 2F, 3B–3D, 4A, 5B–5H, 6B, 6D, 6F, S1A, S1B, S2A, S2B and S3. (XLSX)
