# Supplementary figures and images for: ASNA1 is essential for cardiac development and function by regulating tail-anchored protein stability and vesicular transport in cardiomyocytes
Source: PLoS Genet. 2025 Dec 10;21(12):e1011964. doi: 10.1371/journal.pgen.1011964 (PMC12694866; doi:10.1371/journal.pgen.1011964)

Figure S1

A

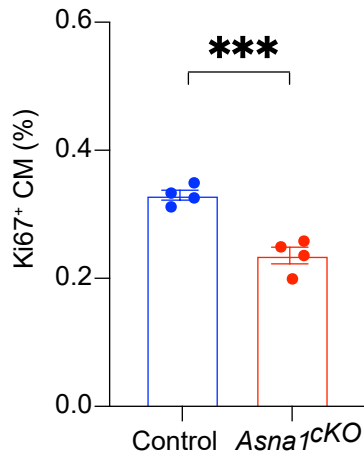

B

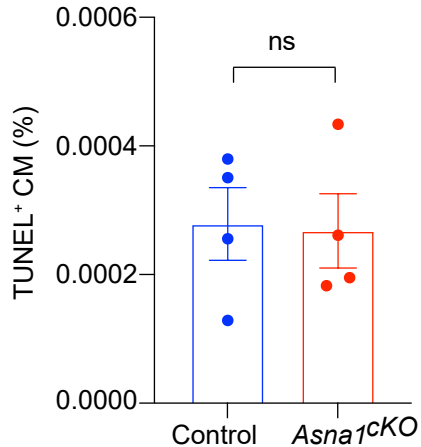

Supplement: S1 Fig — (A) Quantification of Ki67 ⁺ cardiomyocytes (CMs) shows a significant reduction in proliferative cells in Asna1cKO hearts compared to controls (***, P < 0.001). (B) Quantification of TUNEL⁺ cardiomyocytes indicates no significant difference in apoptosis between control and Asna1cKO groups (ns, not significant). Data are presented as mean ± SD, with individual data points shown. (PDF) [file pgen.1011964.s001.pdf]

Figure S2

A

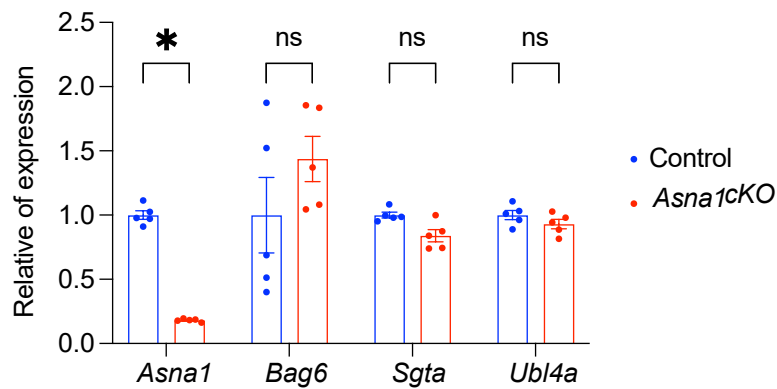

B

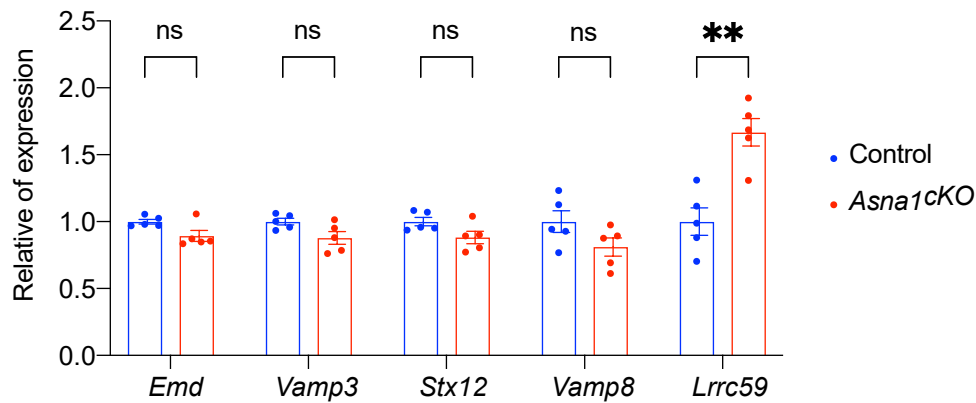

Supplement: S2 Fig — (A) Relative mRNA expression levels of genes involved in the transmembrane recognition complex (TRC) pathway (Asna1, Bag6, Sgta, Ubl4a) were assessed by qPCR in E14.5 embryonic hearts from control (blue) and Asna1cKO (red) mice. (B) Relative mRNA expression levels of selected tail-anchored (TA) protein-encoding genes (Emd, Vamp3, Stx12, Vamp8, Lrrc59) were measured from control (blue) and Asna1cKO (red) hearts. Expression values were normalized to housekeeping genes and are shown relative to control. Each dot represents an individual biological replicate; bars indicate mean ± SD. (PDF) [file pgen.1011964.s002.pdf]

Figure S3

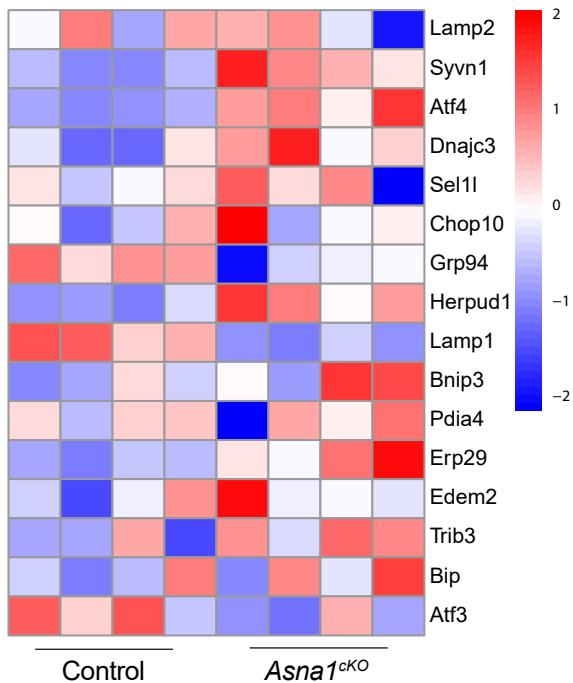

Supplement: S3 Fig — No significant differences in gene expression were observed between Asna1cKO and control hearts. (PDF) [file pgen.1011964.s003.pdf]
