# Supplementary material for: ASNA1 is essential for cardiac development and function by regulating tail-anchored protein stability and vesicular transport in cardiomyocytes
Source: PLoS Genet. 2025 Dec 10;21(12):e1011964. doi: 10.1371/journal.pgen.1011964 (PMC12694866; doi:10.1371/journal.pgen.1011964)
Supplement: S1 Table — (PDF) [file pgen.1011964.s004.pdf]

Supplementary Table S1. Genotype distribution of Asna1 conditional knockout mice across developmental stages

Stage	Asna1$^{f/f}$;Cre-	Asna1$^{f/+}$;Cre-	Asna1$^{f/+}$;Cre+	Asna1$^{f/f}$;Cre+
E14.5	10	9	9	12
E16.5	9	10	12	11
E17.5	15	13	16	12
E18.5	5	7	6	4(live)1(dead)
P0	18	13	13	5(dead)
P1	4	4	5	0
P21	17	20	19	0
