# Supplementary material for: ASNA1 is essential for cardiac development and function by regulating tail-anchored protein stability and vesicular transport in cardiomyocytes
Source: PLoS Genet. 2025 Dec 10;21(12):e1011964. doi: 10.1371/journal.pgen.1011964 (PMC12694866; doi:10.1371/journal.pgen.1011964)
Supplement: S2 Table — (PDF) [file pgen.1011964.s005.pdf]

Supplementary Table S2. Antibodies Used in this study

Antibody	Vendor	Catalogue number	Species	Working concentration
ASNA1	LifeSpan Biosciences	LS-C133055	Mouse	1:2000 (WB)
SGTA	Proteintech	11019-2-AP	Rabbit	1:2000 (WB)
UBL4A	Proteintech	14253-1-AP	Rabbit	1:2000 (WB)
TRC35	Novus Biologicals	NBP1-86732-25ul	Rabbit	1:2000 (WB)
CAML	Proteintech	23327-1-AP	Rabbit	1:2000 (WB)
WRB	Synaptic systems	324 002	Rabbit	1:2000 (WB)
Junctophilin-2 / JPH2	Santa Cruz	sc-377086	Mouse	1:2000 (WB)
Dysferlin / DYSF	Abcam	ab124684	Rabbit	1:2000 (WB)
Emerin / EMD	Santa Cruz	sc-15378	Rabbit	1:2000 (WB)
TMPO	Santa Cruz	sc-28541	Rabbit	1:2000 (WB)
STX5	Proteintech	26711-1-AP	Rabbit	1:2000 (WB)
STX12	Proteintech	14259-1-AP	Rabbit	1:2000 (WB)
SEC61β	Proteintech	15087-1-AP	Rabbit	1:2000 (WB)
VAPA	Proteintech	15275-1-AP	Rabbit	1:2000 (WB)
VAPB	Proteintech	14477-1-AP	Rabbit	1:2000 (WB)
VAMP3	Proteintech	10702-1-AP	Rabbit	1:2000 (WB)
VAMP8	Abcam	ab76021	Rabbit	1:2000 (WB)
LRRC59	Sigma	HPA030829-100UL	Rabbit	1:2000 (WB)
GAPDH	Santa Cruz	sc-377086	Mouse	1:5000 (WB)
Donkey anti mouse	Invitrogen	A21202	Donkey	1:3000 (WB)
Donkey anti rabbit	Invitrogen	A31572	Donkey	1:3000 (WB)
