# Supplementary material for: ASNA1 is essential for cardiac development and function by regulating tail-anchored protein stability and vesicular transport in cardiomyocytes
Source: PLoS Genet. 2025 Dec 10;21(12):e1011964. doi: 10.1371/journal.pgen.1011964 (PMC12694866; doi:10.1371/journal.pgen.1011964)
Supplement: S3 Table — (PDF) [file pgen.1011964.s006.pdf]

## Supplementary Table S3. Primer sequences used for qPCR in this study

| Gene Name | Forward Primer | Reverse Primer |
|---|---|---|
| Asna1 | CAGCCACCAGGCCAATCATA | CTGGTTGGGCCTCCTTCTTG |
| HSPA5 (BiP) | TCAGCCAATTATCAGCAAACTCT | TTTTCTGATGTATCCTCTTCACCAGT |
| Grp94 | CTGCTGTGTCCTGCTGACCTT | TGCGTTTAACCCATCCAACTGA |
| Dnajc3 | CACAGTTTCACGCTGCAGTT | CTCTGTAGTCTTGCGGCAGT |
| Ero1lb | GAAGCGTTCATTGACTGGGC | GCCCTTGTAGCCAGTGTACC |
| Pdia4 | CTCCGGAAAGCTTGGCTGTT | GGTGCATAGAACTCCAGTAGCA |
| Herpud1 | AGCCAATCAGAACTTGCGGA | GGCCTCTGTCTGAACGGAAA |
| Edem2 | ACATCCGAGTGGTAGGAGGA | GCTGTACAGGTAACAGGGGT |
| Sel1l | GAAGTGGGGCTTCTGCGAAA | TTGGTGTGATTCATGCCTGC |
| Syvn1 | ACGCTGCTCCTCTTCCTCA | ACCATGGTCATCAGAATGGCG |
| Atf4 | ATCTCAGTGTGGATCTCGGT | GCTCTCCCTGTAGACGCTTC |
| DDIT3 (Chop) | GCAGCGACAGAGCCAGAATA | CAAGGTGAAAGGCAGGGACT |
| Atf3 | CCAGCCACAGTCTCACTCAG | GACCTGGCCTGGATGTTGAA |
| Trib3 | CTTTTGGAACGAGAGCAAGGC | GTCCCCATGGGTCTTCGTG |
| Bnip3 | GTCGCCTGGCCTCAGAAC | AGATTCATGCTGGGCATCCA |
| Lamp1 | GCCCTGGAATTGCAGTTTGG | TGCTGAATGTGGGCACTAGG |
| Lamp2 | AGGAGCCGTTCAGTCCAATG | GTGTGTCGCCTTGTCAGGTA |
| Nppa (ANF) | GATAGATGAAGGCAGGAAGCCGC | AGGATTGGAGCCCAGAGTGGACTAGG |
| Nppb (BNF) | AAGTCCTAGCCAGTCTCCAGA | GAGCTGTCTCTGGGCCATTTC |
| Myh6 | CTGCTGGAGAGGTTATTCCTCG | GGAAGAGTGAGCGGCGCATCAAGG |
| Myh7 | TGCAAAGGCTCCAGGTCTGAGGGC | GCCAACACCAACCTGTCCAAGTTC |
| Col1a1 | TCACCAAACTCAGAAGATGTAGGA | GACCAGGAGGACCAGGAAG |
| Col3a1 | ACAGCAGTCCAACGTAGATGAAT | TCACAGATTATGTCATCGCAAAG |
| Bag6 | CAATGCCTGGTGTCCCTGCTTT | GCTTGCTGTTCCACCAGAAGGA |
| Sgta | TGGAGGACAGTGACCTTGCTCT | TCTTCAGAGGGTGGTGTCCTGT |
| Ubl4a | CGTCACTTCAGTGTAGCAGATGC | CCATAGCCTCAGTCACTTCAGG |
| Emerin | GGGACCTCACTTGTAGATGCTG | GATGCTCTGGTAGGCACTGTCT |
| Vamp3 | AGACCAGAAGCTCTCGGAGCTA | ACCAGGACACTGATCCCTATCG |
| Vamp8 | TGGACCACCTCCGAAACAAG | TTAGGGAGGTCACCCCTGAG |
| Stx12 | AGCCAATGTGGAAAGCTCGGAG | TCACTGACAGGACAAGCACGAG |
| Lrrc59 | ATCATGACCAAGGCCGGTAG | GTGAGGCCACAGAAATCCGA |
| Vapb | GAAGGTGATGGAAGAGTGCAGG | GCTGTTGCTCGGCATCGCCTT |
| Sec61b | TGCGGGATCCACTGTTCGGCA | GGAATCTTCCGTGTAGAATCGCC |
| Gapdh | CACAGTCAAGGCCGAGAATGGGAA | GTGGTTCACACCCATCACAAACATG |
| Polr2a | CGAGAAGGTCTCATTGACACGG | ACCACCTGGTTGATGGAGTTCC |
